# Supplementary material for: Tolerance of Combined Drought and Heat Stress Is Associated With Transpiration Maintenance and Water Soluble Carbohydrates in Wheat Grains
Source: Front Plant Sci. 2020 Oct 15;11:568693. doi: 10.3389/fpls.2020.568693 (PMC7593570; doi:10.3389/fpls.2020.568693)
Supplement: Supplementary file 1 [file Data_Sheet_1.docx]

| Name | Pedigree | Year of release | Origin |
| --- | --- | --- | --- |
| Currawa | Northern-Champion/Cretan//Little-Club[56][37][39][1138][1451] | 1912 | Australia |
| Odessa ES19565 | Not available | 1930 | Ethiopia |
| Koda | Dundee/Kenya-745-C-6042//Bobin*2/Gaza[39][1138]; Dundee/Gabo[56] | 1955 | Australia |
| Mendos | Spica/Koda//Gabo/3/(Sib)Mengavi[39][117][1138] | 1964 | Australia |
| Synthetic W7984 | Altar-84(TR.DR)/(TR.TA)CI-18[1540] |  | Mexico |
| Frame | Molineux/3*Dagger[1866][2854] | 1997 | Australia |
| Young | VPM-1/3*Beulah//Silverstar[3810] | 2005 | Australia |
| Gladius | (DH)RAC-875/Krichauff//Excalibur/Kukri/3/RAC-875/Krichauff/4/RAC-75//Excalibur/Kukri[3794] | 2007 | Australia |

**Supplementary table S1.** Origins and pedigrees of the eight wheat genotypes used in the study.

| Genotype | Part | WW/D | D/D&H | WW/D&H |
| --- | --- | --- | --- | --- |
| Currawa | Grains | 0.18 | 0.47 | 0.71 |
|  | Spike | **0.05** | 0.31 | **0.01** |
|  | Stem | 0.42 | 0.35 | 0.99 |
| Odessa | Grains | 0.55 | 0.38 | 0.89 |
|  | Spike | **0.03** | 0.98 | **0.06** |
|  | Stem | 0.44 | 0.19 | 0.78 |
| Koda | Grains | 0.73 | 0.32 | 0.7 |
|  | Spike | 0.15 | 0.78 | **0.06** |
|  | Stem | 0.11 | 0.59 | 0.41 |
| Mendos | Grains | 0.34 | 0.87 | 0.15 |
|  | Spike | 0.13 | 0.89 | **0.06** |
|  | Stem | 0.25 | 0.86 | **0.10** |
| Synthetic | Grains | 0.14 | **0.03** | 0.47 |
|  | Spike | 0.19 | 0.27 | **0.02** |
|  | Stem | 0.64 | **0.05** | 0.15 |
| Frame | Grains | 0.52 | 1.00 | 0.55 |
|  | Spike | 0.55 | 0.50 | 0.13 |
|  | Stem | **0.08** | 0.45 | **0.02** |
| Young | Grains | 0.86 | 0.97 | 0.95 |
|  | Spike | 0.99 | 0.99 | 1.00 |
|  | Stem | 0.91 | 0.97 | 0.98 |
| Gladius | Grains | 0.75 | 0.45 | 0.85 |
|  | Spike | 0.50 | 0.65 | 0.17 |
|  | Stem | 0.96 | 0.32 | 0.23 |
|  | | | | |

**Supplementary table S2.** Statistical significance of differences in percentages of water-soluble carbohydrates in different parts of wheat plants: stem, grains and spike, between WW and D, D and D&H, or WW and D&H. In bold: p < 0.1.

(A)

**
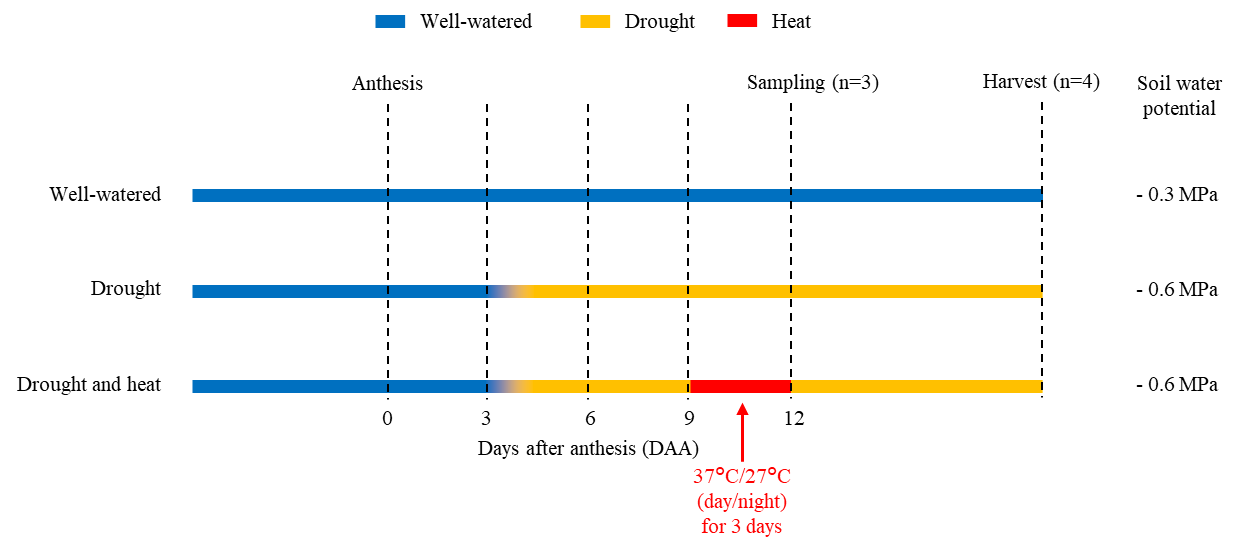
**

(B)

**
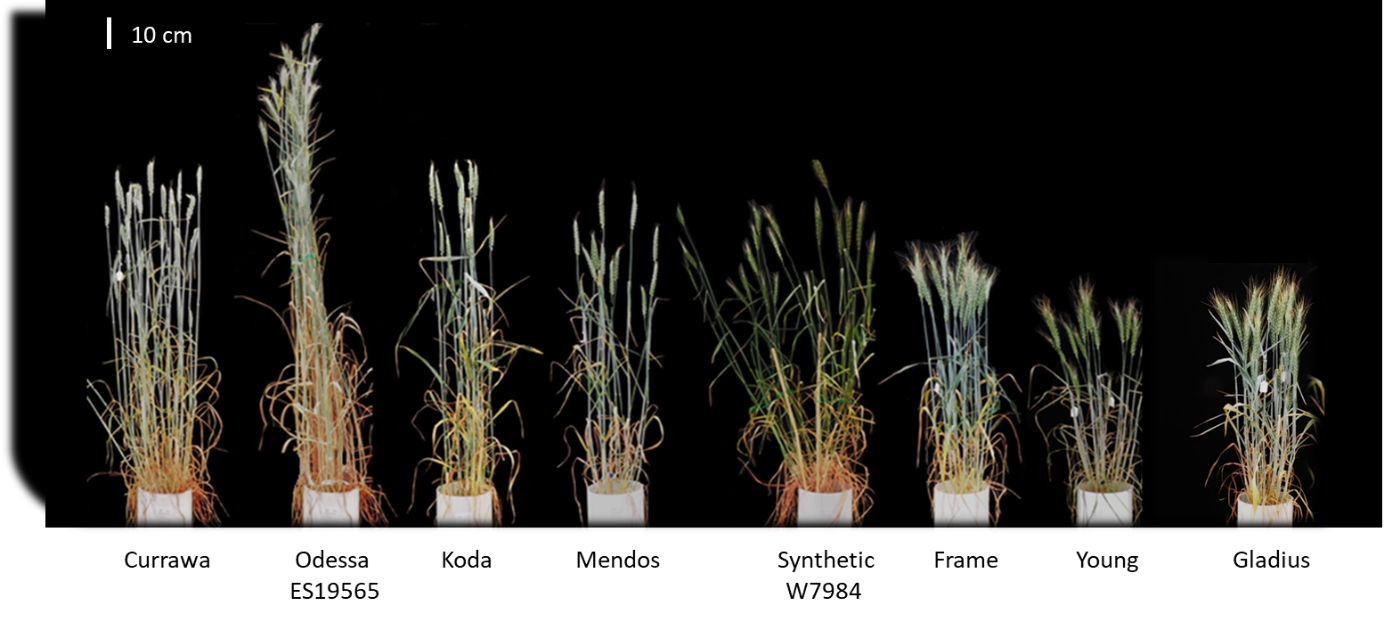
**

Currawa Odessa Koda Mendos Synthetic Frame Young Gladius

ES19565 W7984

Old genotypes Modern genotypes

**Supplementary figure S1.** (A) Schematic of the treatment design. Plants were well watered (soil water potential (SWP) = -0.3 MPa) until anthesis. Drought (SWP = -0.6 MPa) was applied 3 days after anthesis (DAA) and maintained until harvest. Three-day heat treatment was applied 9 DAA. Three replicates per genotype and per treatment were sampled 12 DAA, and four replicates were harvested. (B) Images of plants of different genotypes following combined D&H stress at 12 DAA.

(A)

**
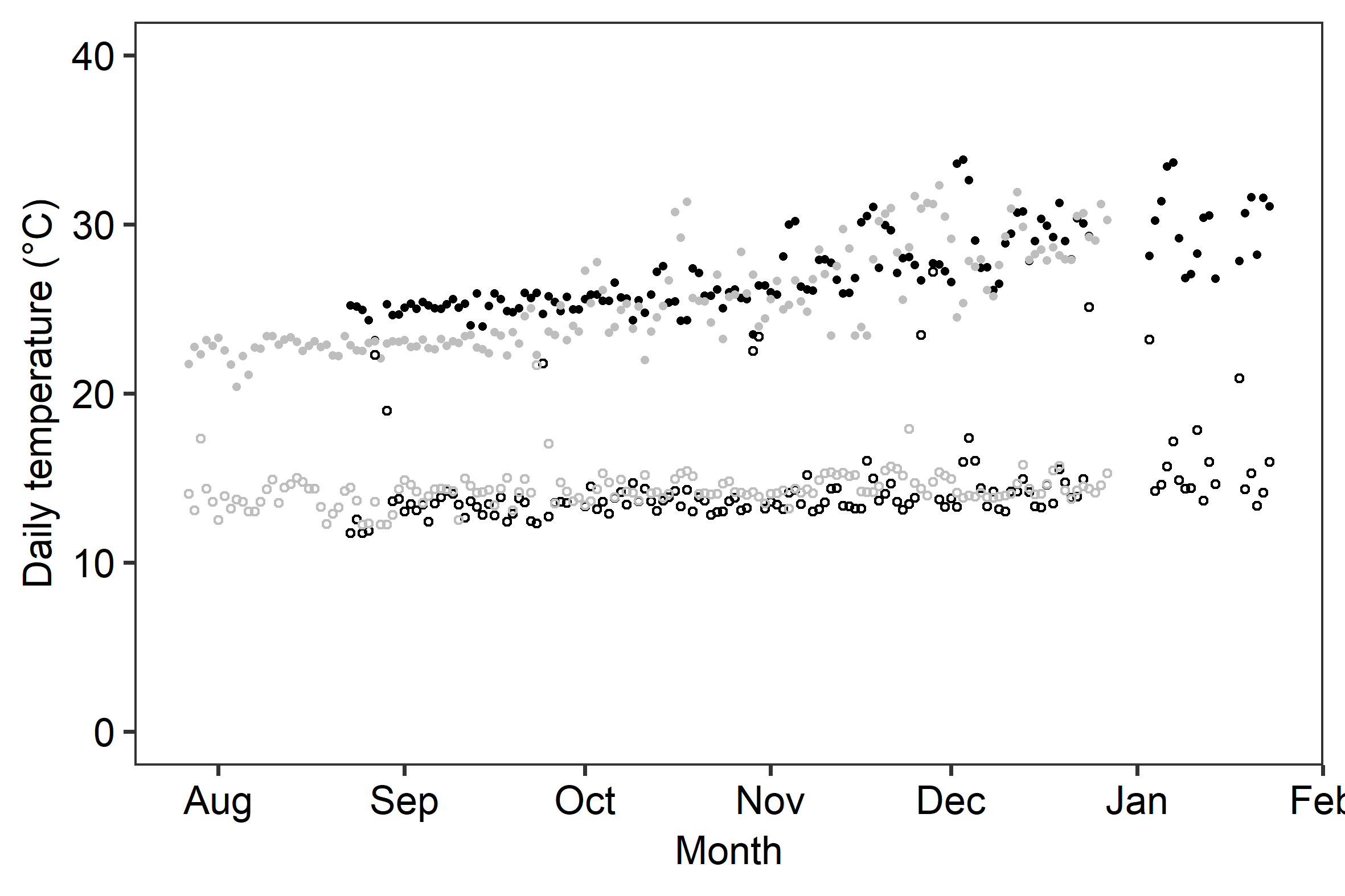
**

(B)

**
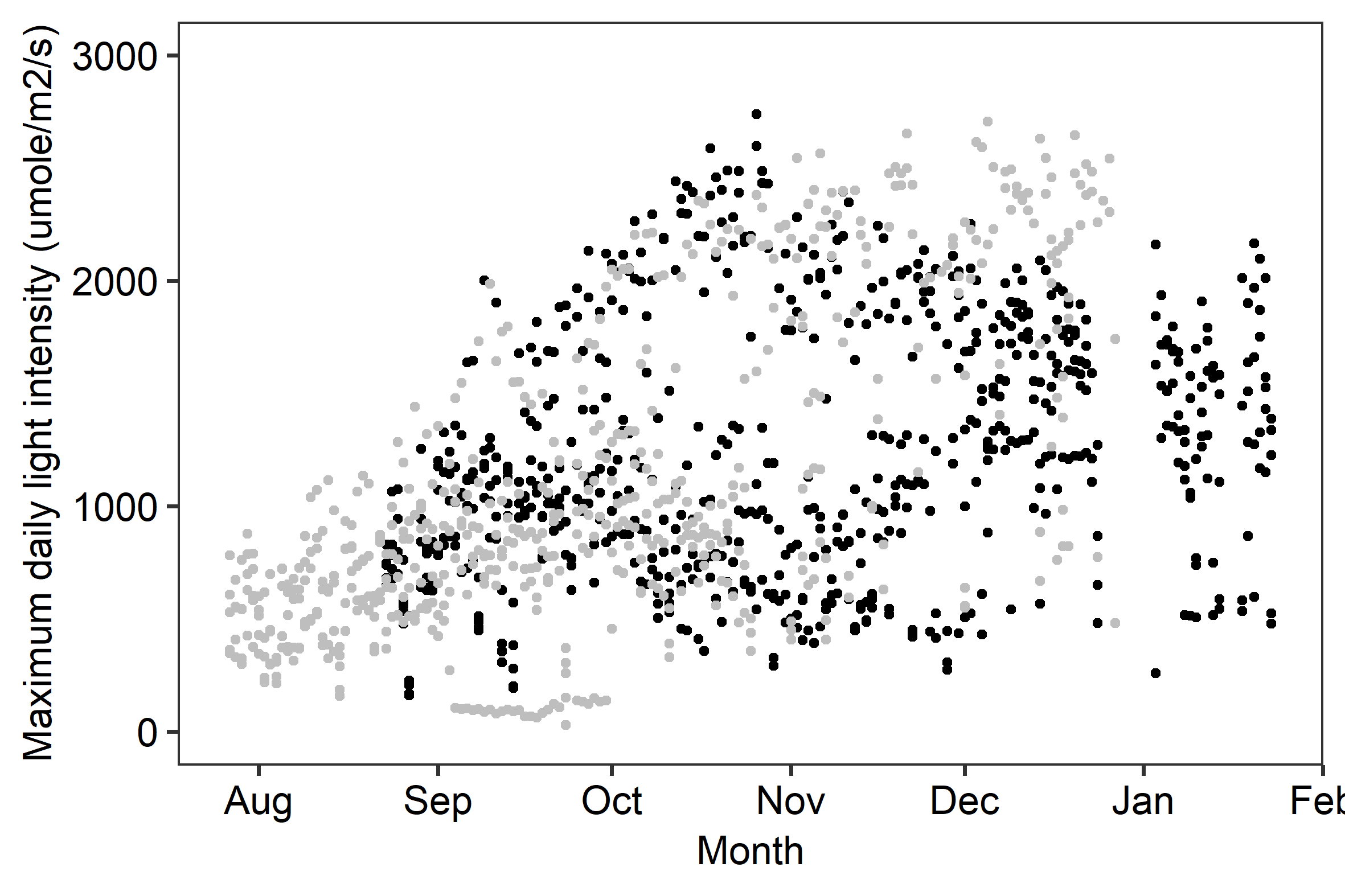
**

(C)

**
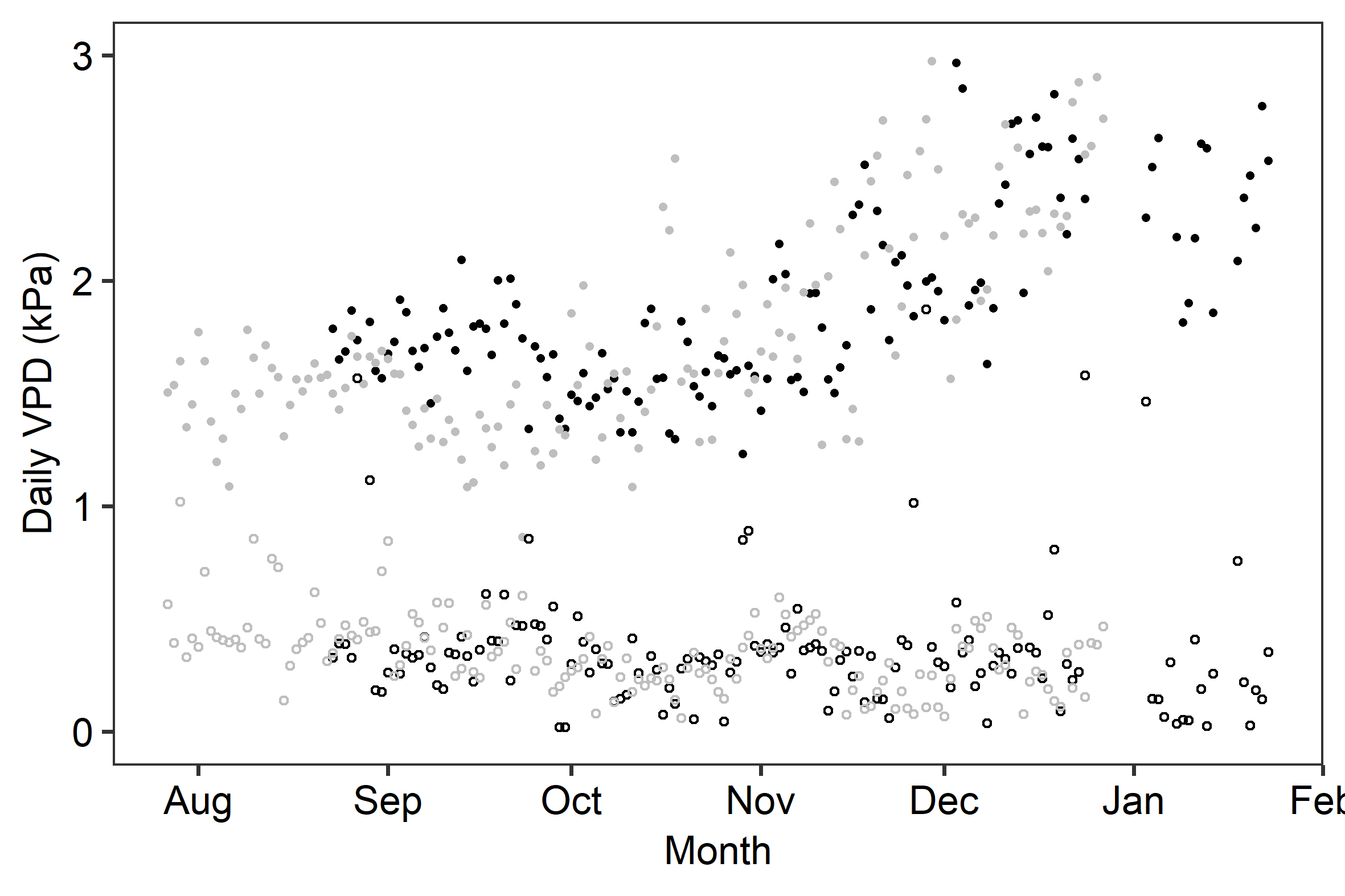
**

**Supplementary figure S2.** Daily maximum and minimum temperature (A), maximum daily light intensity (B), and daily maximum and minimum VPD (C) in the glasshouse experiments in 2016 (in grey) and 2017 (in black). Maximum values are shown in filled points; minimum values are shown in empty points.


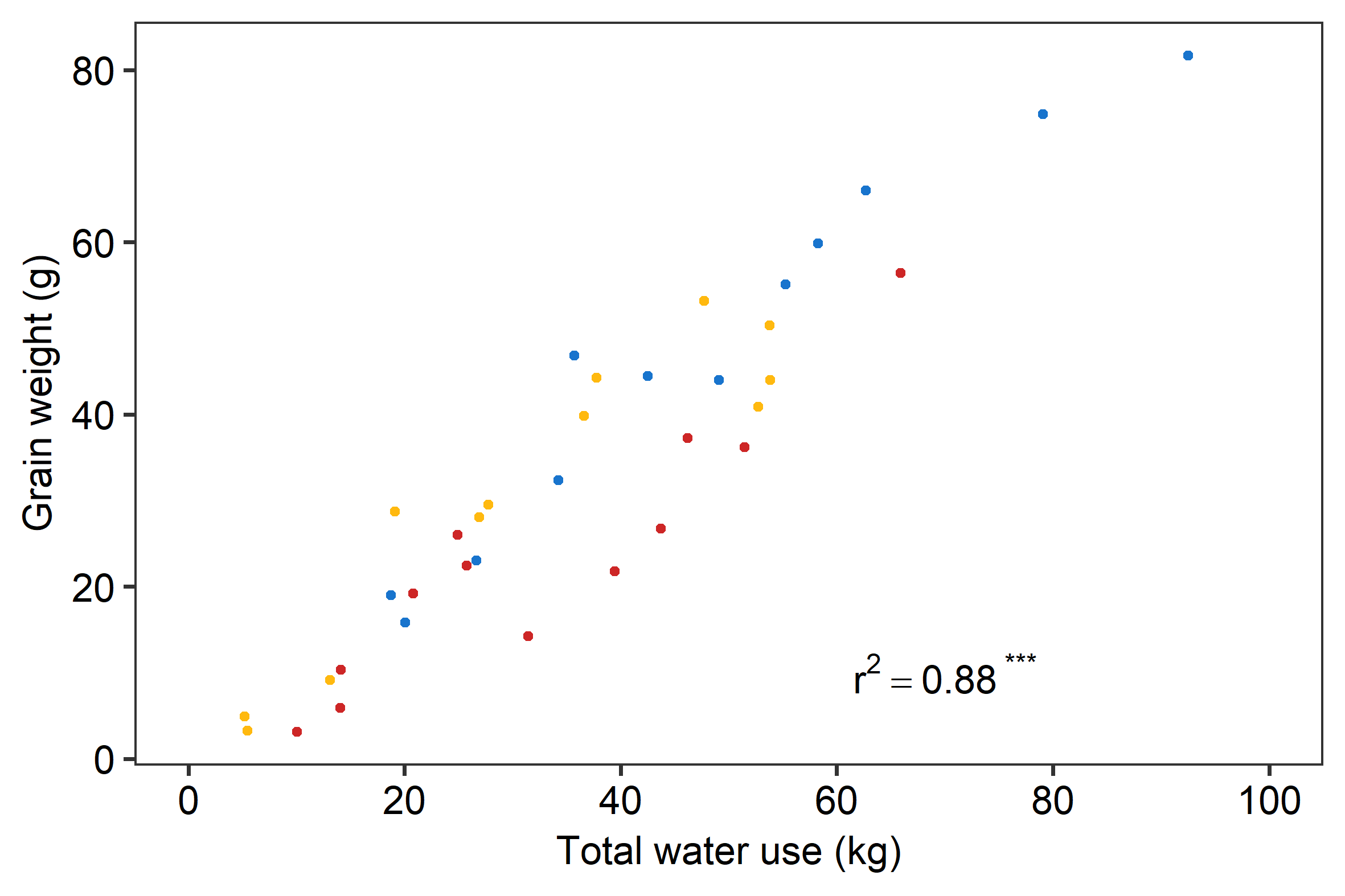


**Supplementary figure S3.** Relationship between total water used and final grain weight per plant (n=3) during Experiment 2 (2017). Each point represents one plant. r^2^ and p-value of the linear regression (‘***’ p < 0.001) are indicated. Plants grown under well-watered conditions (blue), drought (orange) or combined drought and heat (red).


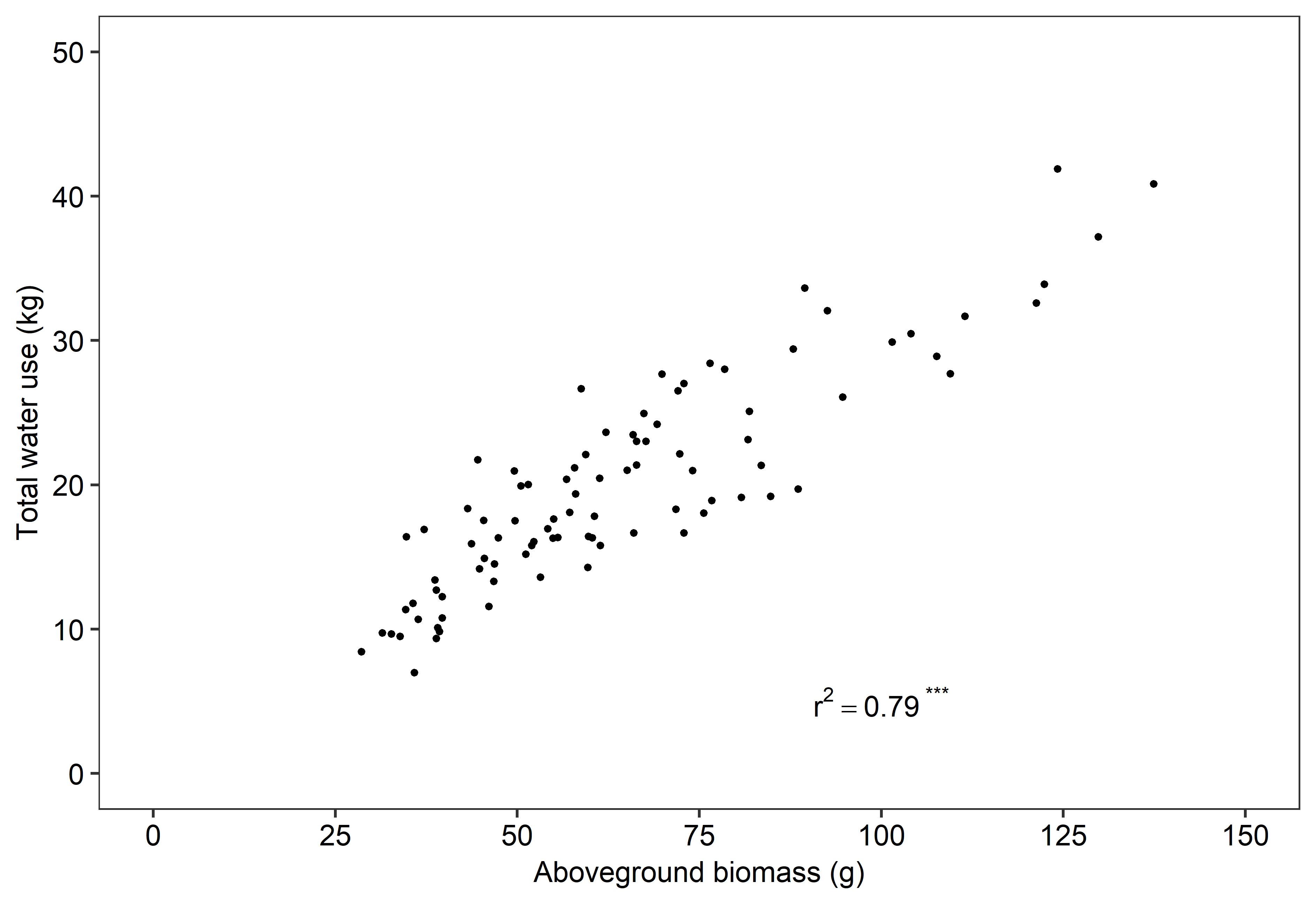


**Supplementary figure S4.** Relationship between aboveground biomass (excluding grains) and total water used. Each point represents one plant. r^2^ and p-value of the linear regression (‘***’ p < 0.001) are indicated.

(A)


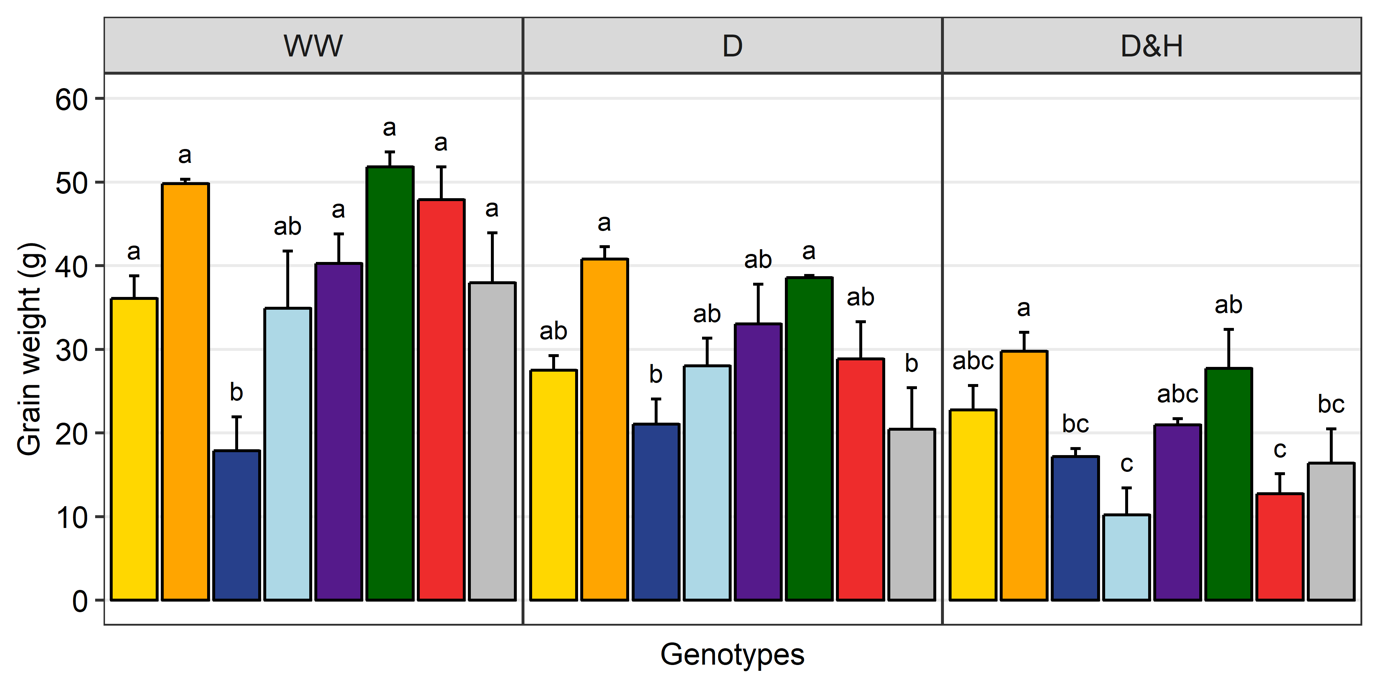


(B)


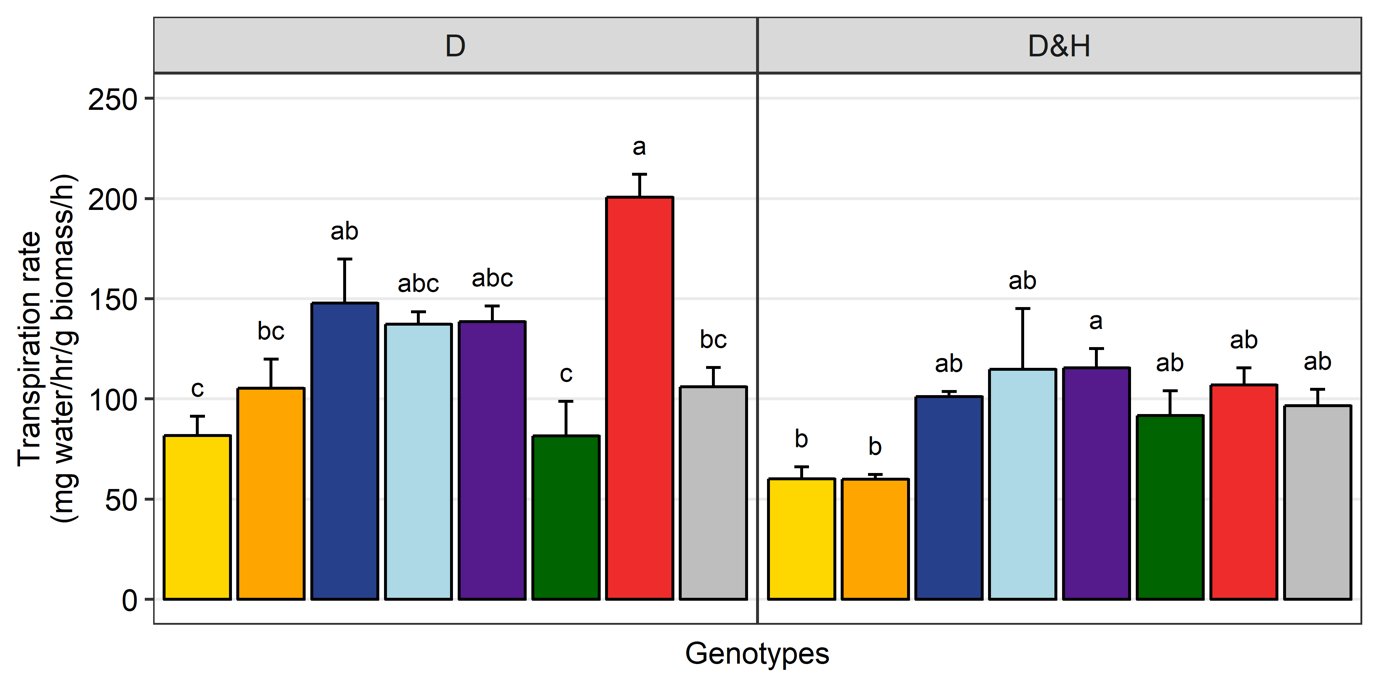


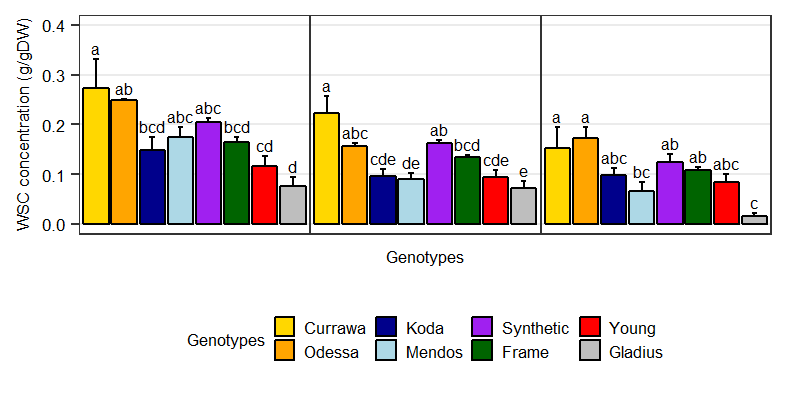


(C)


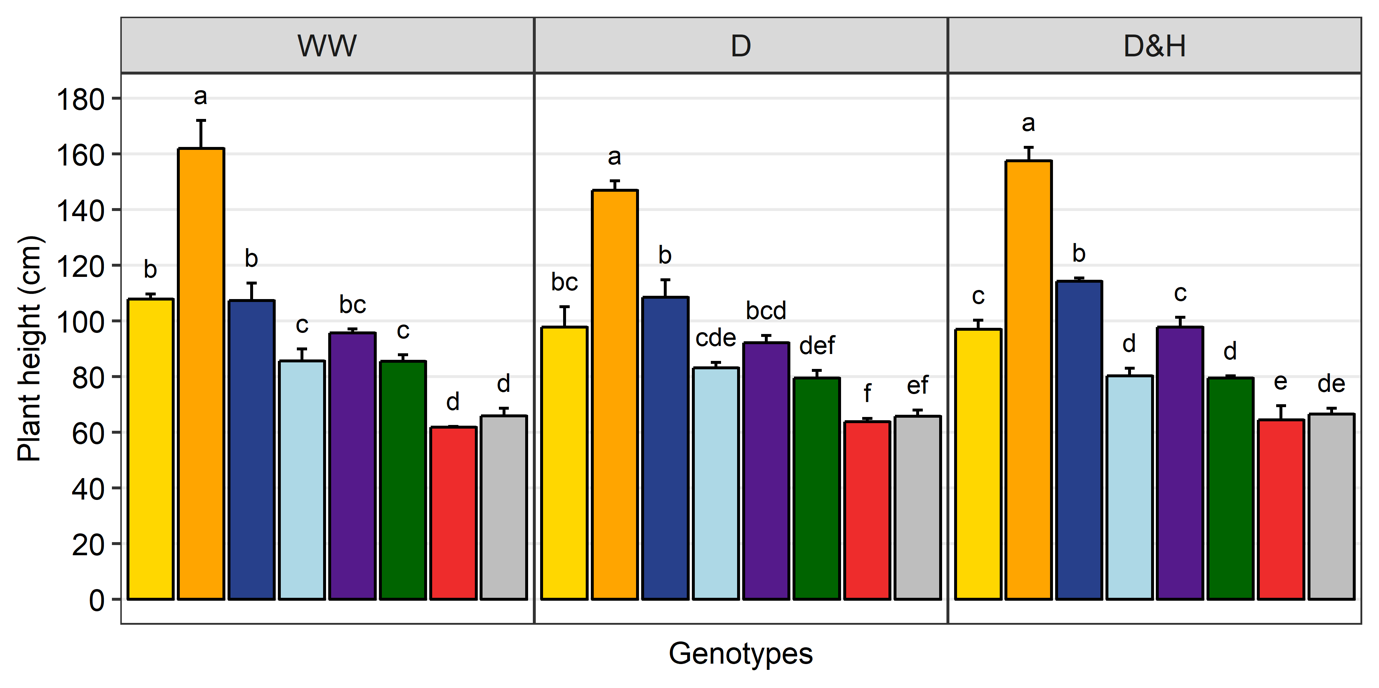


(D)


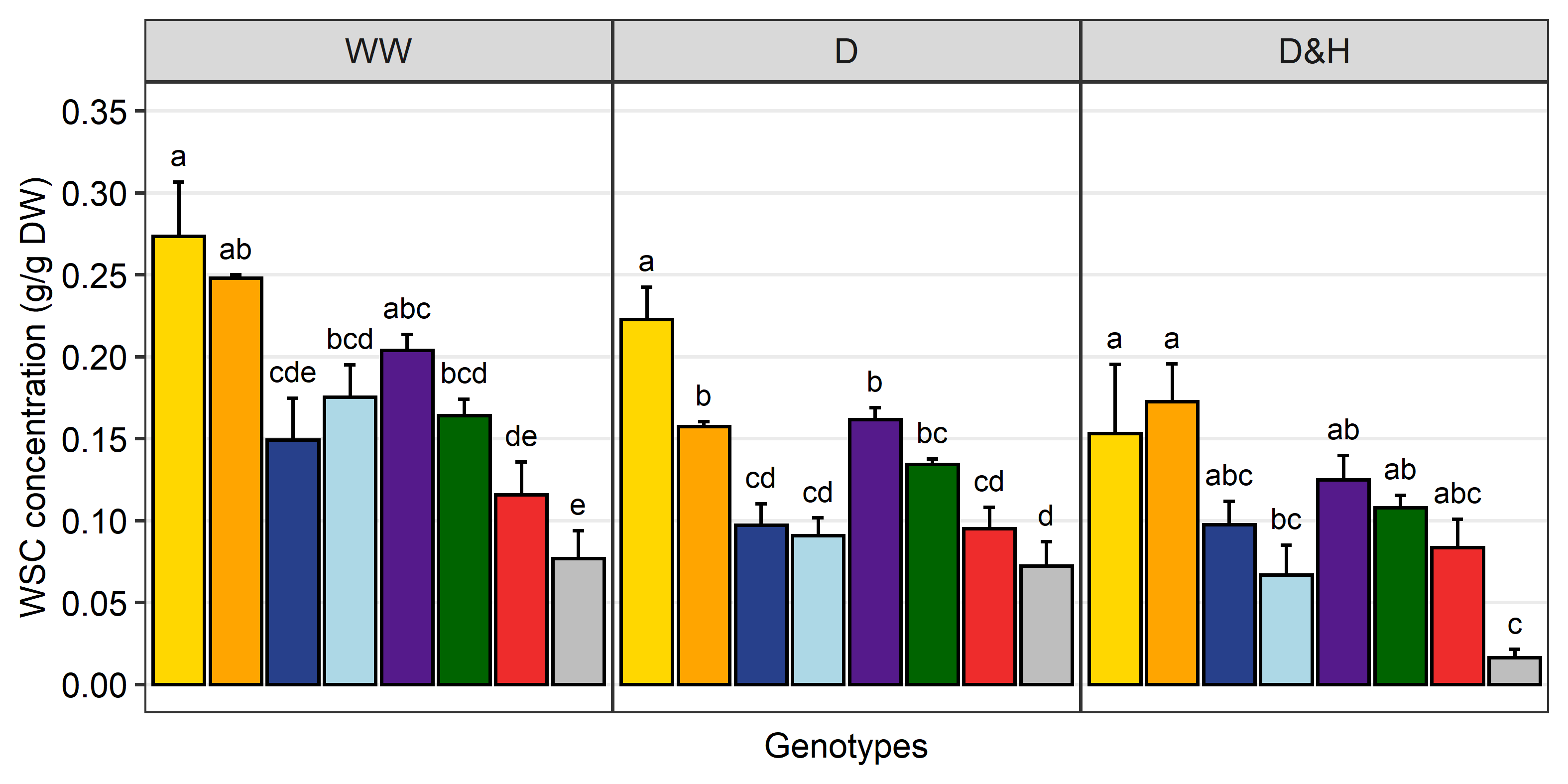


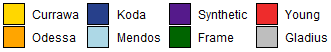


**Supplementary figure S5.** Genotypic differences in final grain weight (A), transpiration rate at VPD = 2 kPa (B), plant height (C) and WSC concentration in the stem (D). Plants grown in well-watered conditions (WW), drought (D) or combined drought and heat stress (D&H). Values are means and standard error of four replicates (n=4). Values represent average per plant. Data were subjected to two-way ANOVA. Letters indicate the results of Tukey’s test comparing genotype effect within each treatment (p < 0.1).
